# Acoustic tweezing of microparticles in microchannels with sinusoidal cross sections

**DOI:** 10.1038/s41598-021-97132-7

**Published:** 2021-09-09

**Authors:** Elnaz Attar Jannesar, Hossein Hamzehpour

**Affiliations:** 1 Department of Physics, K.N. Toosi University of Technology, Tehran, 15875-4416 Iran; 2 School of Physics, Institute for Research in Fundamental Sciences (IPM), Tehran, 19395-5531 Iran

**Keywords:** Biological physics, Condensed-matter physics, Fluid dynamics

## Abstract

Acoustic tweezing of bioparticles has distinct advantages over other manipulation methods such as electrophoresis or magnetophoresis in biotechnological applications. This manipulation method guarantees the viability of the bio-particles during and after the process. In this paper, the effects of sinusoidal boundaries of a microchannel on acoustophoretic manipulation of microparticles are studied. Our results show that while top and bottom walls are vertically actuated at the horizontal half-wave resonance frequency, a large mono-vortex appears, which is never achievable in a rectangular geometry with flat walls and one-dimensional oscillations. The drag force caused by such a vortex in combination with the tilted acoustic radiation force leads to trapping and micromixing of microparticles with diameters larger and smaller than the critical size, respectively. Simulation results in this paper show that efficient particle trapping occurs at the intermediate sinusoidal boundary amplitudes. It is also indicated that in a square-sinusoidal geometry there are two strong vortices, instead of one vortex. Sub-micrometer particles tend to be trapped dramatically faster in such a geometry than in the rectangular-sinusoidal ones.

## Introduction

Acoustophoresis has been studied extensively over the past decades as a nondestructive^1,2^, precise and label-free^3,4^ methodology using ultrasound standing waves^5^ to manipulate microparticles and biological cells based on their intrinsic properties i.e. size, density and compressibility in lab-on-a-chip and microfluidic systems^6^. Some examples of manipulated particles are: circulating tumor cells^3,4^, yeast cells^2^, raw milk^7^, lipids^8^, erythrocytes^9^, bacteria^10–12^ and stem cells^13,14^. Micro-scale acoustical devices have a variety of applications in biotechnology, pharmacology, drug delivery, clinical diagnostics, food analysis and forensic sciences^6,15^. These techniques make a large series of tasks possible, such as synchronization^16^, enrichments^3,17^, sorting by size^18–22^, size-insensitive sorting^23^, separation from the container^7–9,24^, patterning or trapping^14,25–28^ and fluid micro-mixing^29–32^.

Two fundamental nonlinear acoustic phenomena are involved in all the above cases and compete with each other to move suspended particles inside a microchannel. First, the acoustic radiation force, caused by the scattering of acoustic waves from the surface of particles, tends to focus particles on the nodal or anti-nodal plane of the acoustic standing waves^33–36^. Then, the Stokes drag force of the acoustic streaming velocity field, caused by viscous stresses in acoustic boundary layers, appears as steady fluid flow vortices which tend to defocus and spread out the suspended particles^37–40^. A critical particle diameter is determined as a crossover from the radiation force dominated region to the acoustic streaming-induced drag force dominated region^41,42^.

The acoustic radiation force depends linearly on particle volume, acoustic energy and contrast factor (a parameter used to determine the contrast between density and compressibility of a particle to the surrounding medium). Particles with diameters larger than the critical diameter are enforced with the acoustic radiation force. On the other hand, the acoustic streaming drag-force depends linearly on the acoustic energy, viscosity of the fluid, radius of particles and a factor related to the channel geometry. Tiny particles smaller than the critical size are affected by the acoustic streaming flows. Any changes in the boundary conditions dramatically affect the shapes of this vortices and therefore on the acoustophoretic motion of tiny particles. This phenomenon can be troublesome in some cases but if treated properly, it can be helpful in patterning or mixing applications^43,44^.

The geometry plays a key role in the shapes of the acoustic streaming patterns. Some biotechnological applications have been reported by several groups, considering geometrical effects. Focusing of sub-micrometer particles and bacteria in the cross-section of a nearly-square microchannel has been reported by Antfolk et al.^10^. A single roll streaming flow has been observed experimentally using two-dimensional acoustic streaming phenomena. Nama et al.^32^ have suggested a microchannel with the sharp-edged sidewalls as a micromixer. Huang et al.^29^ have optimized the design of such sharp-edged microchannels and reported that mixing performance varies at different frequencies and tip angles . Shortly afterward, a programmable microfluidic pump has been established using oscillations of tilted sharp-edge structures^31^. Ahmed et al.^45^ have found that acoustically driven sidewall-trapped microbubbles would act as a fast microfluidic mixer. As another application for cavitation microstreaming, Yazdi and Ardekani^12^ have shown that microbubbles inside a horseshoe structure are able to trap bacterial aggregations. Attempts have also been made to theoretically investigate the resonance frequencies of liquid-gas interface trapped over horseshoe-shaped structures and compare with experimental observations^46^. A theoretical model has been proposed^47^ to quantify the resonant frequencies and viscous dissipation factors for water-air bubbles trapped in the circular microcavities. Further investigation^48^ has shown that microstreaming induced by air-water membranes on defended oscillating membrane equipped structures (DOMES) leads to an active micromixer on lab-on-a foil devices. As the micron scale surface profiles dramatically affect the inner streaming vortices, Lei et al.^49^ have performed a numerical study on the effects of the roughness of the fluid channels rather than being perfectly flat. Sinusoidally shaped, semi-circular, triangular, square, and trapezoidal surfaces were included. The effects of sinusoidal boundaries of a microchannel on acoustic streaming patterns have studied by Jannesar and Hamzehpour^50^ and a classification of some repetitive patterns have reported.

There are many experimental methods for fabrication of microchannels^51,52^. Soft lithography techniques, especially rapid prototyping and replica molding are some appropriate methods to design arbitrary geometries. In rapid prototyping, a design is first established in a computer-aided design program (CAD), then cast on PDMS materials.

In our study, creation of a single strong dominant vortex in the cross section of a microchannel with sinusoidal boundaries in one-dimensional oscillatory condition is numerically studied using a very simple mechanism. Such a vortex can never be produced in rectangular geometry with flat boundaries using one-dimensional oscillations. Also, the trajectory of several particles inside the cross section is simulated to investigate the functionality of the system at different frequencies for special curvatures in the cross-section of the microchannel.

The rest of the paper is organized as follows. In “Theoretical background” section the theoretical background is presented. This is followed in “Numerical model and boundary conditions” section by a description on the numerical model. The results are presented and discussed in “Results and discussion”. Some conclusions are stated in the last section.

## Theoretical background

In the absence of external body forces and heat sources, there are three important governing equations in the microfluidic systems as^53,54^

1a $$\begin{aligned}&\partial _t{\rho }={\varvec{\nabla }}\cdot [-\rho \varvec{v}], \end{aligned}$$

1b $$\begin{aligned}&\partial _t{(\rho \varvec{v})}={\varvec{\nabla }}\cdot [\varvec{\sigma }-\rho \varvec{vv}], \end{aligned}$$

1c $$\begin{aligned}&\partial _t{(\rho \varepsilon +\tfrac{1}{2}\rho v^2)}={\varvec{\nabla }}\cdot [k^{th}{\varvec{\nabla }}T+\varvec{v}\cdot \varvec{\sigma }-\rho (\varepsilon +\tfrac{1}{2} v^2)\varvec{v}] . \end{aligned}$$

The continuity Eq. 1a expresses conservation of mass where the mass density is $\rho$, the Navier–Stokes equation 1b expresses conservation of momentum where momentum density is $\rho \varvec{v}$, and the heat-transfer equation 1c expresses conservation of energy where energy density is $\rho (\varepsilon +\tfrac{1}{2} v^2)$. Noteworthy, $\varepsilon$ is internal energy per unit mass, *T* is the temperature field, $\varvec{v}$ is the velocity fluid in the medium and $k^{th}$ is the thermal conductivity. Also, $\varvec{\sigma }$ is the stress tensor of the fluid (Cauchy stress tensor) as

2 $$\begin{aligned} \varvec{\sigma }=\varvec{\tau }-p\varvec{I}=\eta [{\varvec{\nabla }}\varvec{v}+({\varvec{\nabla }}\varvec{v})^\text {T}]+[(\eta ^B-\tfrac{2}{3}\eta ){\varvec{\nabla }}\cdot \varvec{v}-p\,]\varvec{I}, \end{aligned}$$

where *p* is the pressure field, $\varvec{I}$ is the unit tensor and $\varvec{\tau }$ is viscous stress tensor, which is expressed in terms of dynamic shear viscosity $\eta$ and bulk viscosity $\eta ^B$.

Thermal effects are neglected in this work, because the thermal boundary layer thickness (thermal diffusion length), $\delta _{t}$, in fluids is much smaller than viscous boundary layer thickness (viscous penetration depth), $\delta _{\nu }$^56^ that are defined as

3a $$\begin{aligned}\delta _{t}=\sqrt{\frac{2D_{th}}{\omega }}, \end{aligned}$$

3b $$\begin{aligned}\delta _{\nu }=\sqrt{\frac{2\nu }{\omega }}, \end{aligned}$$

where $D_{th}$ is the thermal diffusion constant, $\omega$ is angular frequency of the acoustic field and $\nu =\frac{\eta }{\rho }$ is the dynamic viscosity.

### First-order perturbation approximation

The initial (zeroth-order) state of the fluid is quiescent, homogeneous, and isotropic. Considering the external acoustic field as a perturbation of the steady state of a fluid, all the fields in standard first-order approximation can be expanded in the form $g=g_0+g_1$ where $g_1$ is much smaller than $g_0$. The perturbation parameter is defined as the dimensionless acoustic Mach number^53^ as

4 $$\begin{aligned} Ma=\frac{\vert \rho _1\vert }{\rho _0}\ll 1, \end{aligned}$$

where $\rho _0$ is the unperturbed density of the fluid and $\rho _1$ is the first order perturbation term of density.

In this study, processes are considered to be adiabatic. As a result, entropy is conserved for any small fluid volume and the acoustic pressure is independent of the first-order fluid temperature. If an acoustic wave constitutes tiny perturbations, the (isentropic) first-order expansion of the equation of state is defined as: $p\left( \rho \right) =p_0+\left( \frac{\partial p}{\partial \rho } \right) _s \rho _1$. This means that the small change in the pressure is related to the small change in the density by $p_1=\left( \frac{\partial p}{\partial \rho } \right) _s \rho _1$. The isentropic compressibility in classical fluid mechanics is defined as $\kappa _s=\frac{1}{\rho }\left( \frac{\partial \rho }{\partial p} \right) _s=\frac{1}{\rho _0 c_s^2}$ where $c_0$ is the speed of sound in water at rest. As a result, in the adiabatic limit we have $\rho _1=\rho _0 \kappa _s p_1$. Additionally, it can be assumed that all first-order fields have a harmonic time-dependent form resulted from an imposed ultrasound field. The first-order equations in the frequency domain become^54,55^

5a $$\begin{aligned}&{\varvec{\nabla }}\cdot \varvec{v}_1-i\omega \kappa _s p_1=0, \end{aligned}$$

5b $$\begin{aligned}&{\varvec{\nabla }}\cdot \varvec{\sigma }_1+i\omega \rho _0\varvec{v}_1=0, \end{aligned}$$

where $\varvec{\sigma }_1$ is given by

6 $$\begin{aligned} \varvec{\sigma }_1=\varvec{\tau }_1-p_1\varvec{I}=\eta _0[{\varvec{\nabla }}\varvec{v}_1+({\varvec{\nabla }}\varvec{v}_1)^\text {T}]+[(\eta _0^B-\tfrac{2}{3}\eta _0){\varvec{\nabla }}\cdot \varvec{v_1}-p_1]\varvec{I}. \end{aligned}$$

Subscript 0 denotes the equilibrium conditions and subscript 1 represents the first-order quantities.

### The time averaged second-order perturbation approximation

Using the second order approximation of the perturbation theory, all the fields can be expressed as $g=g_0+g_1+g_2$. Then, the time averaged second-order perturbation approximation of governing equations are^54,55^

7a $$\begin{aligned}&{\varvec{\nabla }}\cdot {\left\langle \varvec{v}_2 \right\rangle }+\kappa _s \left\langle \varvec{v}_1\cdot {\varvec{\nabla }}p_1\right\rangle =0, \end{aligned}$$

7b $$\begin{aligned}&{\varvec{\nabla }}\cdot [\left\langle \varvec{\sigma }_2\right\rangle -\rho _0\left\langle \varvec{v}_1\varvec{v}_1\right\rangle ]=0. \end{aligned}$$

Note that the stress tensor of the fluid using the second-order perturbation theory is given by

8 $$\begin{aligned} \begin{aligned} \left\langle \varvec{\sigma }_2\right\rangle&=\left\langle \varvec{\tau }_2\right\rangle -\left\langle p_2\right\rangle \varvec{I} =\left\langle \eta _0\left[ {\varvec{\nabla }}\varvec{v}_2+({\varvec{\nabla }}\varvec{v}_2)^\text {T}\right] \right\rangle \\&\quad +\left\langle \left[ \left( \eta _0^B-\tfrac{2}{3}\eta _0\right) \left( {\varvec{\nabla }}\cdot \varvec{v_2}\right) \varvec{I}\right] \right\rangle -\left\langle p_2\right\rangle \varvec{I}, \end{aligned} \end{aligned}$$

### Acoustophoretic forces acting on a small particle in a viscous fluid

A suspending spherical particle with the diameter of *a*, in the long-wavelength limit, $\delta _{\nu },a\ll \lambda$ (i.e. particle size and viscous boundary layer are much smaller than the wavelength of the imposing acoustic wave, $\lambda$) encounters with two acoustophoretic forces.

The the time-averaged radiation force, which appears as a result of scattering of the sound wave on the particles, is given by^56,57^

9 $$\begin{aligned} {\varvec{F}_{{rad}}}=-\pi a^3\left[ \frac{2\kappa _s}{3}Re[f_1^*p_1^{*in}{\varvec{\nabla }}p_1^{in}]-\rho _0 Re[f_2^*\varvec{v}_1^{*in}\cdot {\varvec{\nabla }}\varvec{v}_1^{in}] \right] , \end{aligned}$$

where $\varvec{v}_1^{in}$ and $p_1^{in}$ are the first-order pressure and velocity fields of the incident acoustic wave evaluated at a particle position. Asterisks denote complex conjugations. The prefactors $f_1$ and $f_2$ are the so-called mono and dipole scattering coefficients. In viscous fluids these are calculated as

10a $$\begin{aligned}&f_1({\tilde{\kappa }})=1-{\tilde{\kappa }},&\text {with} \ {\tilde{\kappa }}=\frac{\kappa _p}{\kappa _s}, \end{aligned}$$

10b $$\begin{aligned}&f_2({\tilde{\rho }},\tilde{\delta _{\nu }})=\frac{2\left[ 1-\Gamma (\tilde{\delta _{\nu }}) \right] ({\tilde{\rho }}-1)}{2{\tilde{\rho }}+1-3\Gamma (\tilde{\delta _{\nu }})},&\text {with} \ {\tilde{\rho }}=\frac{\rho _p}{\rho _0}, \end{aligned}$$

10c $$\begin{aligned}&\Gamma (\tilde{\delta _{\nu }})=-\frac{3}{2}\left[ 1+\text {i}\left( 1+\tilde{\delta _{\nu }} \right) \right] \tilde{\delta _{\nu }},&\text {with}\ \tilde{\delta _{\nu }}=\frac{\delta _{\nu }}{a}, \end{aligned}$$

where $\kappa _p$ and $\rho _p$ are the compressibility and density of the particles.

The acoustic streaming flow field $\left\langle \varvec{v}_2\right\rangle$ is caused by the attenuation of the acoustic wave inside a microchannel due to the boundary effects. The time-averaged streaming-induced drag force on a spherical particle with the radius of *a*, moving with the velocity of $\varvec{u}$ far from the channel walls, is given by

11 $$\begin{aligned} {\varvec{F}_{drag}}=6\pi \eta a (\left\langle \varvec{v}_2\right\rangle -\varvec{u}). \end{aligned}$$

The crossover from the dominance of each force is defined through a critical particle radius. For a spherical particle inside a rectangular microchannel, the crossover diameter is

12 $$\begin{aligned} 2a_c=\sqrt{12\frac{\Psi }{\Phi }}\delta _{\nu }, \end{aligned}$$

where $\Psi$ is a factor related to the channel geometry. The acoustophoretic contrast factor is given by $\Phi ({\tilde{\kappa }},{\tilde{\rho }},\tilde{\delta _{\nu }}) =\frac{1}{3}f_1({\tilde{\kappa }})+\frac{1}{2}f_2^{r}({\tilde{\rho }},\tilde{\delta _{\nu }})$, that contains material parameters. The monopole scattering coefficient $f_1$ is real valued and depends only on the compressibility ratio ${{\tilde{\kappa }}}$. The viscosity-dependent dipole scattering coefficient $f_2$ is in general a complex-valued number, and its real value is abbreviated as $f_2^r({\tilde{\rho }},\tilde{\delta _{\nu }})=Re\left[ f_2({\tilde{\rho }},\tilde{\delta _{\nu }})\right]$.

If the radius of the particle becomes less than the critical size, streaming flow effects will become more significant and the radiation force will be suppressed. Otherwise, for particles larger the crossover limit, radiation force will be dominant and make particles move towards the pressure nodes or anti-nodes due to their contrast factor.

## Numerical model and boundary conditions

**Figure 1:**
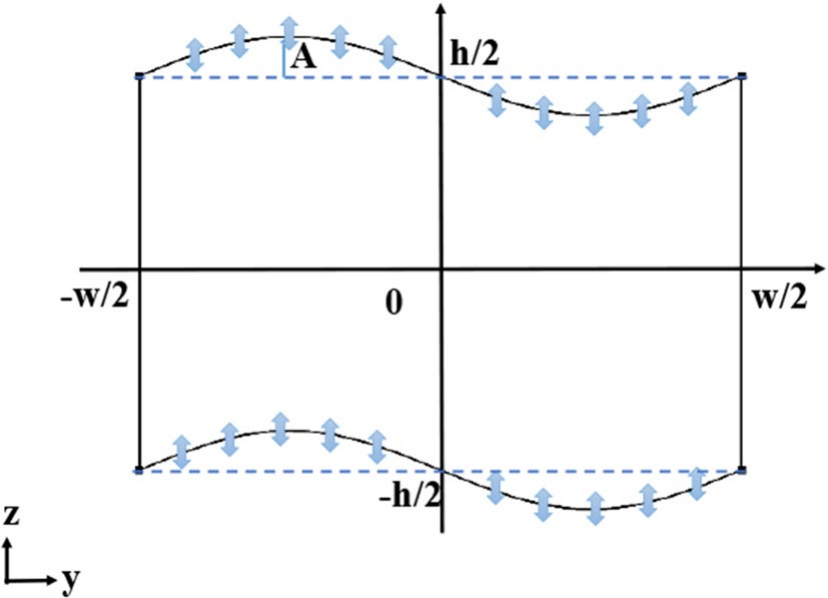
Schematic illustration of a sinusoidal microchannel cross-section in *yz* plane. Blue arrows show the oscillation direction of the actuated boundary walls. Hypothetical basic rectangular microchannel with height of *h* and width of *w* is shown by dashed lines.

As shown in Fig. 1, a microchannel with a rectangular cross-section of the height, $h=100 \;\mu$m and width $w=150\;\mu$m in the *yz* plane is considered, with the top and bottom walls having sinusoidal shapes given by

13 $$\begin{aligned} z =\pm (h/2) - A \sin ({2\pi y}/{w})\;, \end{aligned}$$

where *A* is the amplitude of the sinusoidal boundaries.

The governing Eqs. (5, 7) are solved using the finite element method. We have used the weak-form-PDE of mathematics for both first and second-order equations. The conducted steps are as follows: First, the flow equations are written as source-free flux formulation, $\varvec{\nabla }\cdot \varvec{J}+ F=0$; Then they are converted to the weak-form; Finally, the weak-form equations are solved by mathematics-weak-form-PDE module.

Considering $\varvec{J}$ as a vector and *F* as a scalar (e.g. Eqs. 5a and 7a), the weak form of a source-free flux equation is given by

14 $$\begin{aligned} \int _{\Omega }\left[ -\varvec{\nabla }{\widetilde{\psi }}\cdot \varvec{J}+{\widetilde{\psi }}F\right] d\varvec{r}=0, \end{aligned}$$

where ${\widetilde{\psi }}$ is a test function.

For $\varvec{J}$ as a tensor and $\varvec{F}$ a vector (e.g. Eqs. 5b and 7b), the weak form of a source-free flux equation is

15 $$\begin{aligned} \int _{\Omega }\left[ -\varvec{\nabla }{\widetilde{\Psi }}_m\cdot \varvec{J}+{\widetilde{\Psi }}_m\cdot \varvec{F}\right] d\varvec{r}=0, \;\;\text {for all }m \end{aligned}$$

where ${\widetilde{\Psi }}_m$ is the *m*th component of a test vector $\Psi$.

In all cases, the zero-flux boundary condition, $\varvec{J} \cdot \varvec{n} =0$, is supposed; where $\varvec{n}$ is the normal vector to the boundary surface. Also, all boundaries are considered as hard walls. Top and bottom walls are actuated by an external acoustic field. Therefore, the boundary conditions of the first-order velocity field are

16a $$\begin{aligned}\text{top-bottom}:v_{y1}=0\;, \quad&v_{z1}=v_{bc}\sin (\omega t)\;, \end{aligned}$$

16b $$\begin{aligned}&\text {left-right}:&v_{y1}=0\;, \quad&v_{z1}=0\;, \end{aligned}$$

where $v_{bc}=\omega d$, $\omega =2 \pi f$, and $d=0.1\;$nm. The displacement of the oscillating walls in the *z* direction, *d*, is small enough to justify the use of the perturbation theory.

A zero-mass-flux boundary condition is considered for the second-order velocity field as

17a $$\begin{aligned}&\text {top-bottom}:&v_{y2}=0\;, \quad&v_{z2}=-\frac{\left\langle \rho _1 v_{z1} \right\rangle }{\rho _0}\;, \end{aligned}$$

17b $$\begin{aligned}&\text {left-right}:&v_{y2}=0\;, \quad&v_{z2}=0\;. \end{aligned}$$

**Figure 2:**
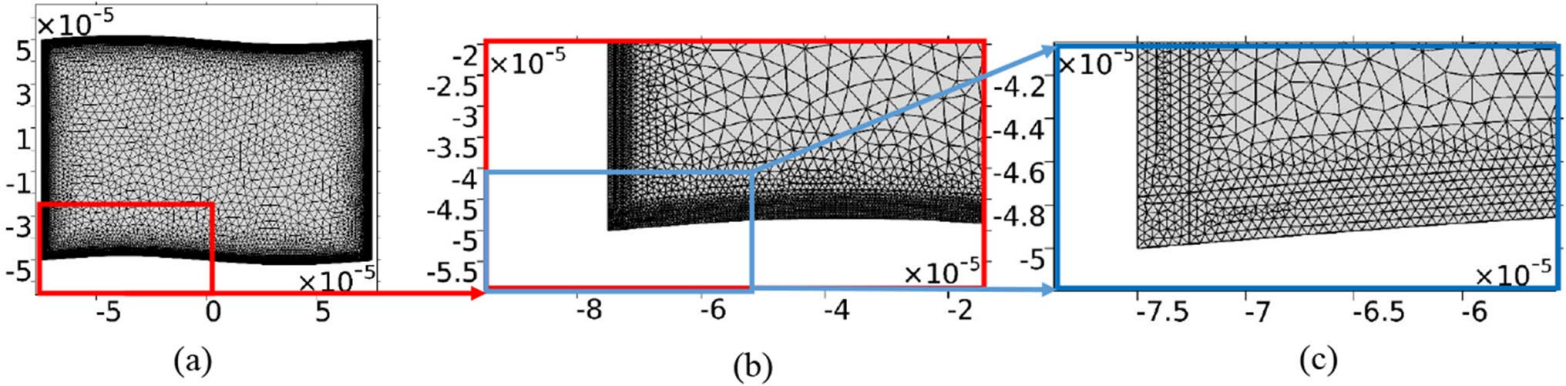
(**a**) Sketch of the spatial mesh of the sinusoidal computational domain in the *yz* plain. (**b**) and (**c**) are two zoom-in scales on the mesh in the lower left corner.

The maximum mesh size in boundaries until 10$\delta _\nu$ and bulk are considered $0.5\mu m$ and $5\mu m$, respectively. In both cases, the mesh element growth rate is 1.3 . Figure 2 presents a sketch of spatial mesh of our computational domain. The fluid inside the microchannel is considered to be quiescent water. Additionally, the physical parameters for water at a temperature of $T= 25^{\circ }\mathrm {C}$ and pressure of $p_0=0.1013$ MPa are presented in Table 1.

**Table 1:** Physical parameters of water at 25 °C and $p_0=0.1013 \;{\mathrm{MPa}}$^54^.

Parameter	Symbol	Value	Unit
Mass density	$\rho _0$	$9.970\times 10^{2}$	kg/m$^3$
Speed of sound	$c_0$	$1.497\times 10^{3}$	m/s
Shear viscosity	$\eta _0$	$8.900\times 10^{-4}$	Pa s
Bulk viscosity	$\eta _0^B$	$2.485\times 10^{-3}$	Pa s

## Results and discussion

Extensive numerical calculations were carried out to study the effects of the geometry of the microchannel walls on two-dimensional acoustophoretic trapping of the microparticles. The results show that the geometry plays a key role in creating a dominant strong vortex of the streaming pattern. The drag force caused by such a vortex inside a microchannel in combination with the tilted acoustic radiation force results in trapping of microparticles with diameters larger than the critical particle diameter. In the following sub-sections we present our results and discuss their implications.

### Creation of a mono-vortex pattern

**Figure 3:**
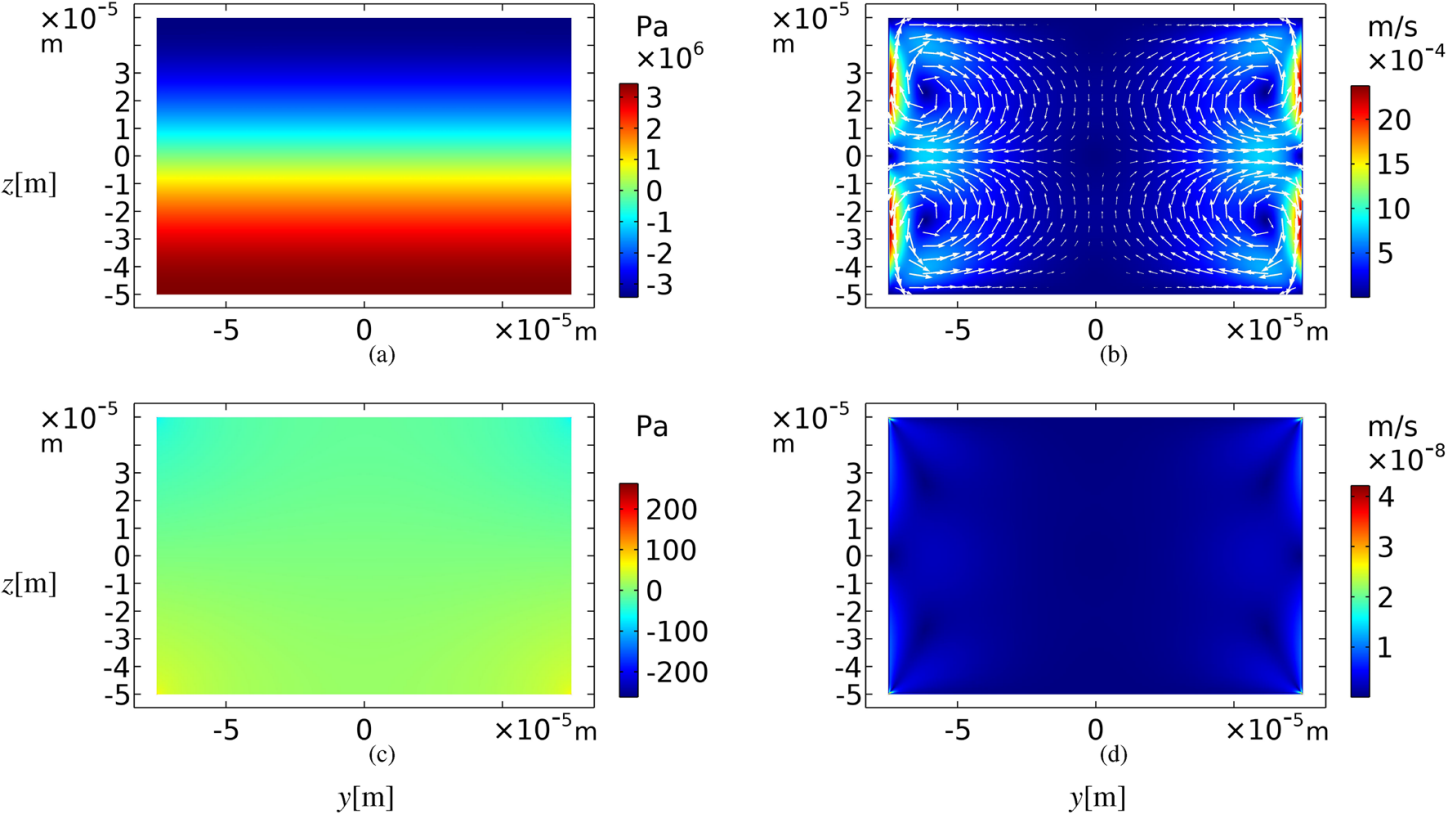
First-order pressure fields (left column) in Pascal and time-averaged second-order velocity fields (right column) in m/s for a rectangular cross-section of a microchannel, while the top and bottom walls oscillation frequency is $f_{v}$ for (**a**) and (**b**), and $f_h$ for (**c**) and (**d**).

In this section our simulation results for a microchannel with sinusoidal cross-section are compared with a rectangular one. The results for the pressure field at the half-wave resonance frequency, i.e. $f_{v}=c_0/2h= 7.48\;{\mathrm{MHz}}$ is shown in Fig. 3a. The actuated boundaries are top and bottom walls and the direction of the oscillation is vertical. At this frequency, eight Rayleigh–Schlichting acoustic streaming vortices are created inside the cross-section of a rectangular microchannel, as expected^57^. Four Rayleigh bulk streaming patterns are distinguishable in Fig. 3b, but four other Schlichting streaming flows inside the boundary layers cannot be recognized in this scale. If the top and bottom boundaries are actuated in the *z* direction with the frequency of $f_{h}=c_0/2w= 5\;{\mathrm{MHz}}$ which is the horizontal half-wave resonance frequency of the microchannel, the creation of acoustic standing waves or acoustic streaming flows are not expected. Figure 3c, d show the first-order pressure field and the acoustic streaming patterns for this frequency in a rectangular cross-section.

As a validity test for our simulations, we have compared some of our results, such as shown in Fig. 3a, b with what was reported by Muller et al.^57^. The results are the same.

**Figure 4:**
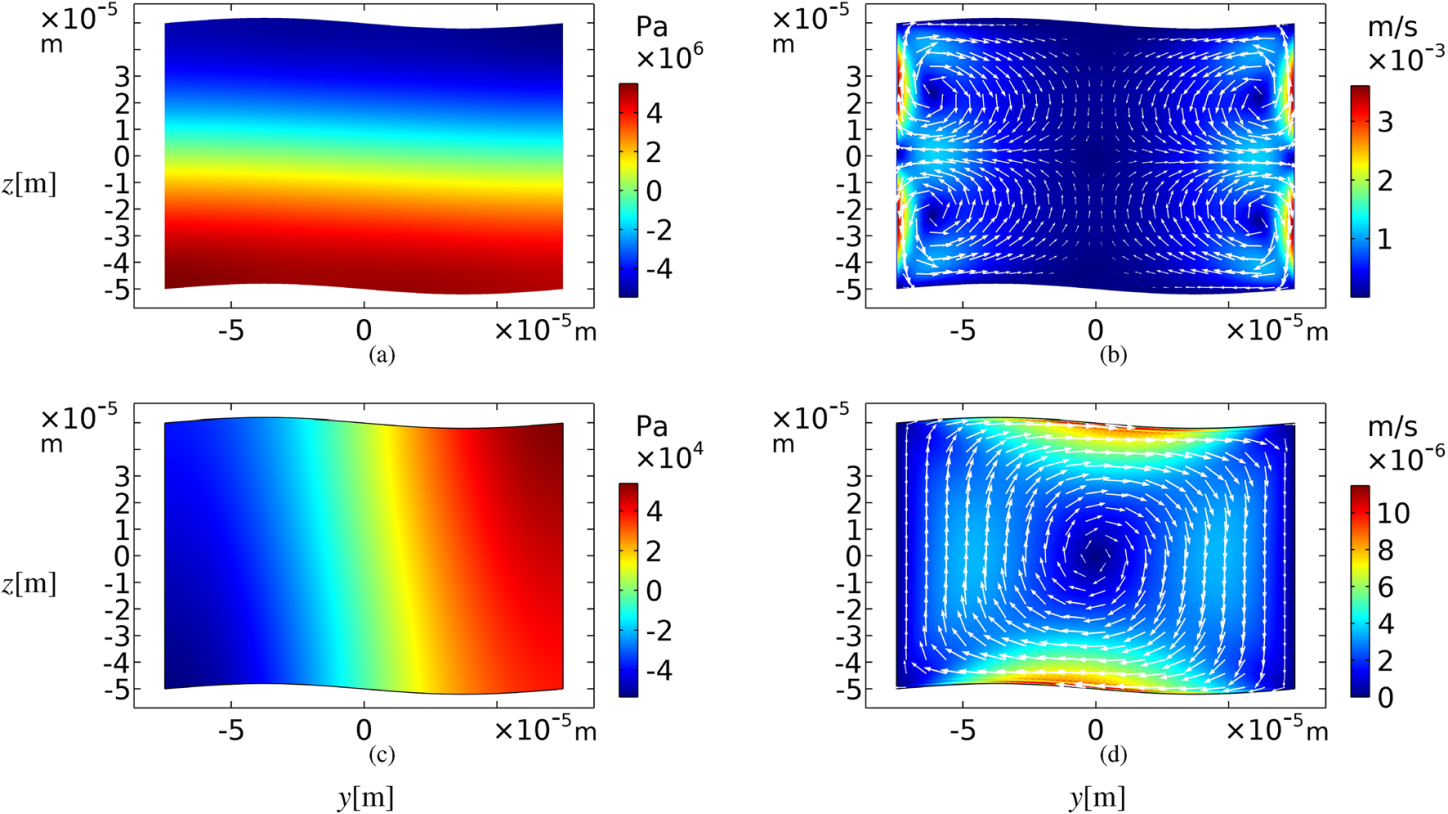
First-order pressure fields (left column) in Pascal and time-averaged second-order velocity fields (right column) in m/s for a sinusoidal yz plane cross-section of a microchannel, while the top and bottom walls oscillation frequency is $f_{v}$ for (**a**) and (**b**), and $f_h$ for (**c**) and (**d**). The amplitude of the sinusoidal boundaries are $A=h/50$.

A different geometry of cross-section, i.e. sinusoidal walls with an amplitude of $A=h/50$ is considered as shown in Fig. 1. Meanwhile, Fig. 4a indicates that at the frequency of $f_{v}$, an approximately vertical standing wave is established. Also, the acoustic streaming pattern presented in Fig. 4b is very similar to Fig. 3b with the same order of velocity magnitudes.

For the horizontal half-wave resonance frequency, $f_{h}$, the results are different from the rectangular case. While the top and bottom walls are oscillating vertically with the frequency $f_h$, a standing acoustic wave is almost created in the horizontal direction as shown in Fig. 4c. In addition, boundary layers appear in the vicinity of the oscillating sinusoidal walls instead of the no-slip fixed boundaries. As shown in Fig. 4d, the resulting acoustic streaming pattern, in this case, is a large mono-vortex. Such a pattern is never achievable in a rectangular geometry with one-dimensional oscillation. A similar vortex has been reported by Antfolk et al.^10^, when applying two-dimensional acoustic actuation to a semi-square cross-section. In their study, all four side walls are involved in the oscillation, whereas in this study, only the top and bottom walls are oscillating in one direction.

**Figure 5:**
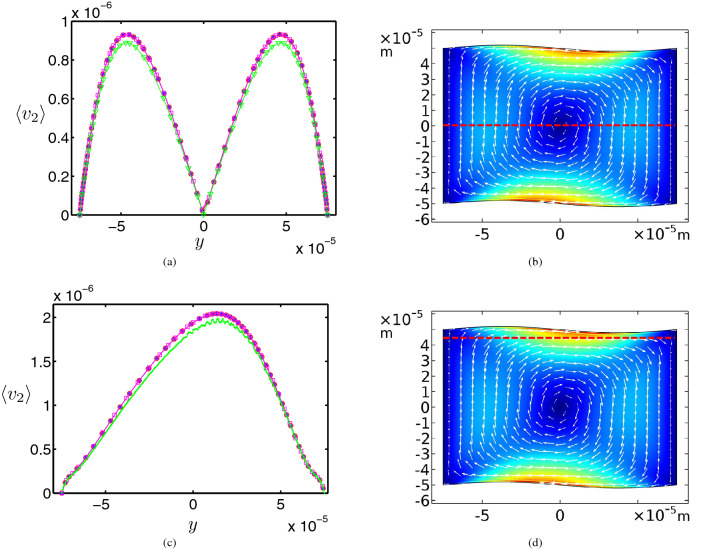
The time-averaged second-order velocity fields $\left\langle \varvec{v}_2 \right\rangle$, (**a**) and (**c**), for four various mesh sizes in two cutlines (**b**) $z=0$ is related to (**a**), and (**d**) $z=+45\;\upmu{\text{m}}$ is related to (**c**). The maximum element mesh sizes are 0.25 and 2.5 ($*$), 0.5 and 5 ($\bigcirc$), 1 and 10 ($\square$), 3 and 30 ($\triangledown$) micrometer for boundary and bulk domains, respectively. The results show that $\left\langle \varvec{v}_2 \right\rangle$ values are converged in finer mesh sizes.

To verify the correctness of the solution, we prove the mesh convergence in our study. As reported by Muller et al.^57^, the time-averaged second-order velocity field $\left\langle \varvec{v}_2 \right\rangle$ converges considerably slower than the first-order fields. So we consider the magnitude of the second-order velocity field for two cutlines, $z=0$ and $z =  + \;45\;\upmu{\text{m}}$, of Fig. 4d with various mesh sizes. The results in Fig. 5 show that $\left\langle \varvec{v}_2 \right\rangle$ values converge for mesh sizes smaller than $1\;\upmu{\text{m}}$ for boundary and $10\;\upmu{\text{m}}$ for bulk domains. In our simulations, we use $0.5\;\upmu{\text{m}}$ and $5\;\upmu{\text{m}}$ for boundary and bulk domains, respectively, which are smaller than the above mentioned values.

**Figure 6:**
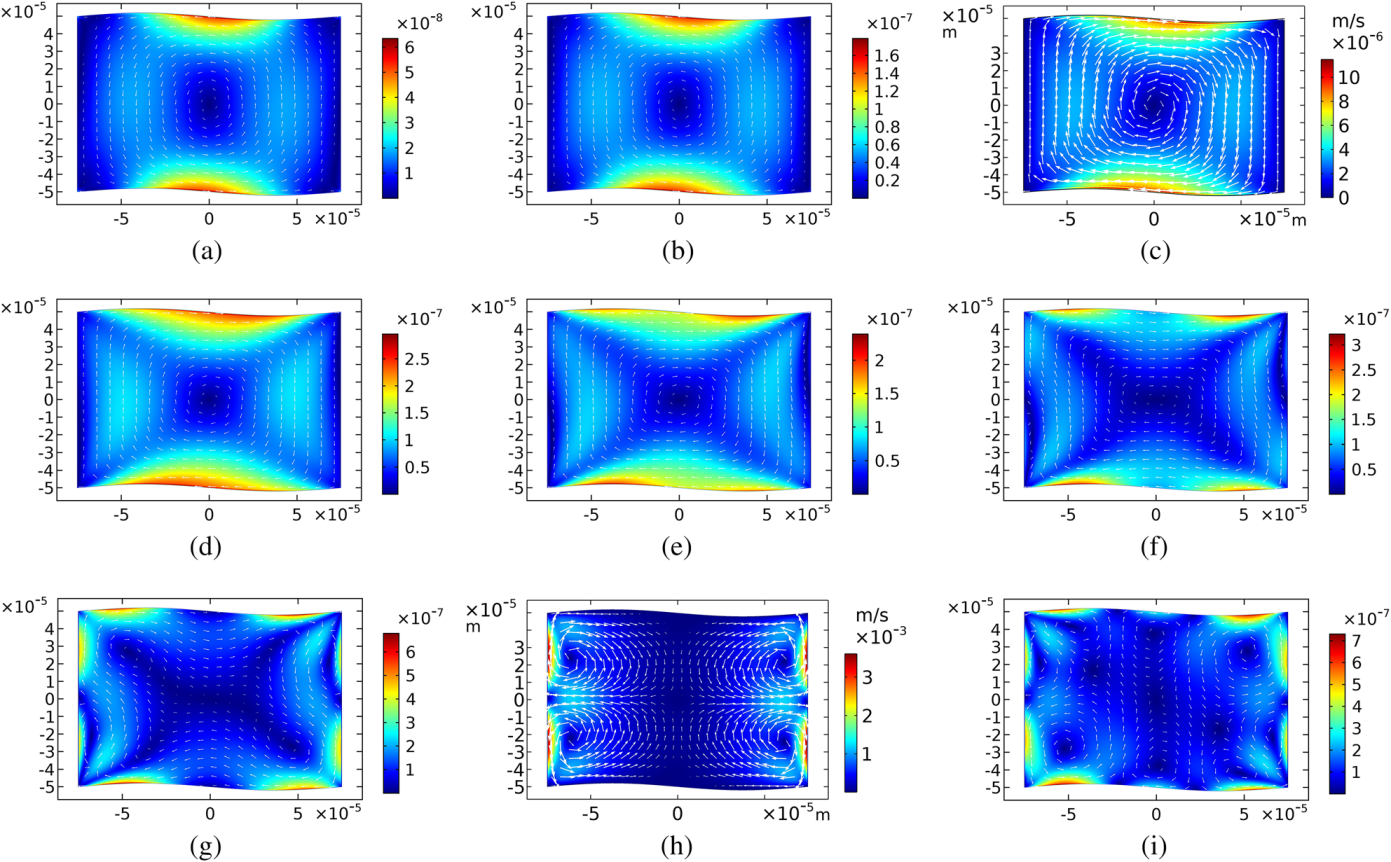
Time-averaged second-order velocity fields,$\langle {\varvec{v}_2} \rangle$, in m/s of fluid for sinusoidal walls with the amplitude of $A=h/50$. Actuation frequencies of the top and bottom walls are (**a**) 4, (**b**) 4.5, (**c**) 5, (**d**) 5.5, (**e**) 6.5, (**f**) 6.5, (**g**) 7, (**h**) 7.5 and (**i**) 8 MHz. Color contours show $\langle {\varvec{v}_2} \rangle$ values and arrows indicate the flow directions.

To study the effects of actuation frequencies on the streaming patterns, some frequencies around two resonance frequencies of the system, $f_h$ and $f_v$, are investigated. The results for frequencies between 4 to 8 MHz are shown in Figs. 6a-i. The mono-vortex patterns appear at the frequencies around $f_h$ (see Fig. 6a-e). Clearly, the strongest one is created at the frequency of 5 MHz.

### Particle tracing through the mono-vortex

**Figure 7:**
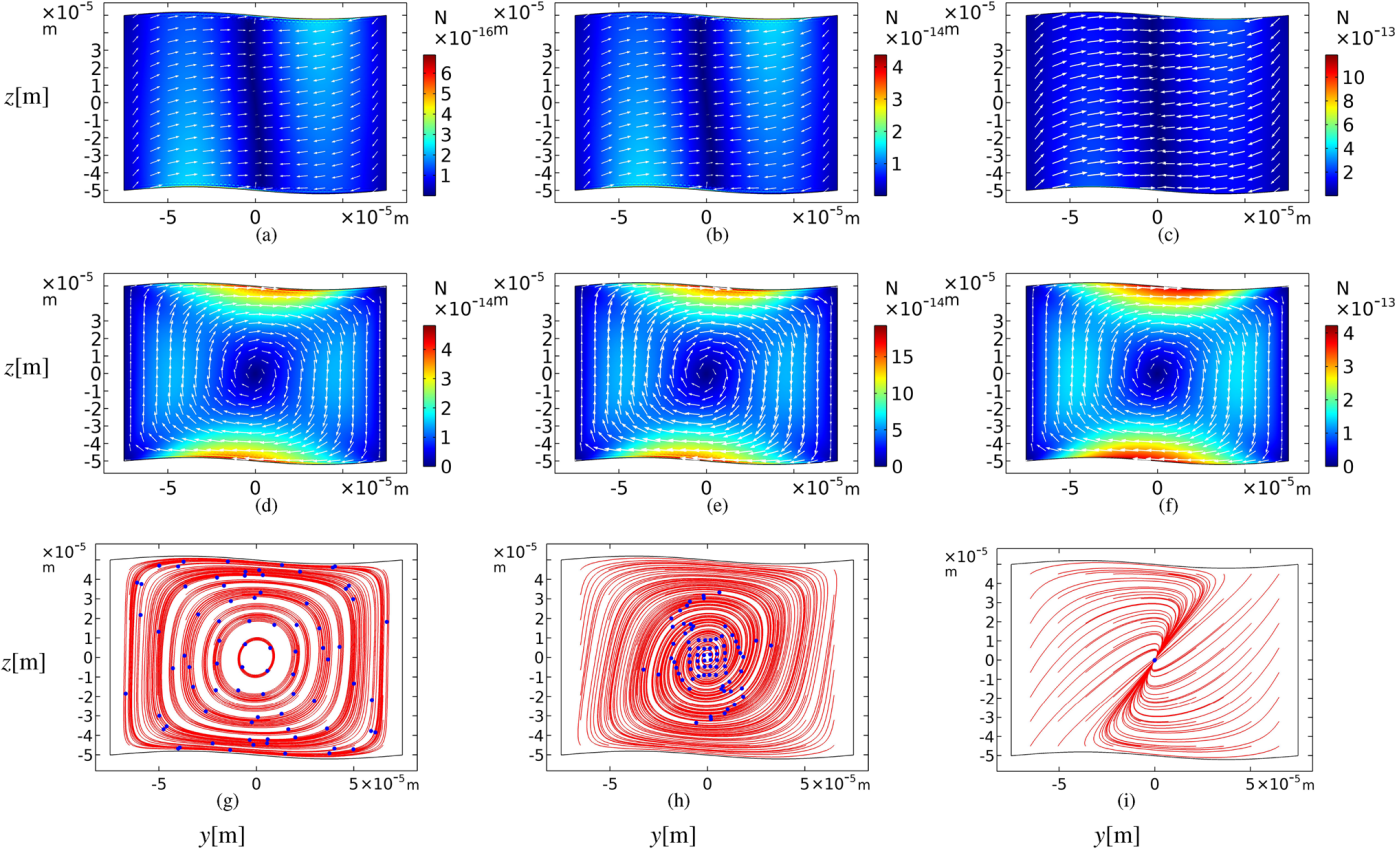
Acoustic radiation force (**a**)–(**c**), acoustic streaming-induced drag force (**d**)–(**f**), both in Newton and particle trajectories (**g**)–(**i**) after 100 s. The amplitude of sinusoidal walls is $A = h/50$ and the actuation frequency of the top and bottom walls is $f_h$. Particle sizes are $d=$ 0.5, 2 and $5\;\mu {\mathrm{m}}$, from left to right. Red lines show 80 particle paths from the beginning and blue points represent the particles.

In this section, 80 polystyrene microparticles are traced inside the sinusoidal microchannel at the actuation frequency of $f_h$. The particles are spreading homogeneously inside the bulk of the domain. The physical properties are shown in Table 2. Figure 7 represents the acoustic radiation and streaming drag force fields and the corresponding particle trajectories after 100 s for various particle sizes. These trajectories are effectively controlled by the strength ratio of two acoustophoretic forces. According to Eqs. (9) and (11), $\varvec{F}_{rad}$ and $\varvec{F}_{drag}$ are functions of $a^3$ and *a*, respectively. The results in Fig. 7 confirm that increasing particle sizes by one order of the magnitude, causes $\varvec{F}_{rad}$ to increases by three order of the magnitude, while $\varvec{F}_{drag}$ increases linearly. Figure 7g-i show that the small particles move in closed paths similar to the streaming pattern and eventually get approximately aligned, while the larger ones tend to move in straight lines towards the pressure nodes (see Fig. 4c for the pressure profile). The reason is that tiny particles are more affected by the acoustic streaming drag force of the fluid flow while large particles are impacted by the acoustic radiation force. The combination of radiation and streaming drag forces at the same order of magnitude causes particles to move in spiral trajectories and finally be trapped in the center of the microchannel.

**Table 2:** Physical parameters of polystyrene particles^57^.

Parameter	Symbol	Value	Unit
Mass density	$\rho _p$	1050	kg/m$^3$
Speed of sound	$c_p$	2350	m/s
Isentropic compressibility	$\kappa _p$	$249\times {10^{-10}}$	$\text {1/Pa}$

The particle size has a significant impact on the trapping time. In all geometries, the critical size is obtained when two acoustic forces are equal. In this study, the viscous boundary layer thickness is ,$\delta _\nu =$ 0.24 $\mu m$, the geometry dependent factor, $\Psi$, is approximately equal to 0.375, which is valid for a planar wall, and the contrast factor is $\Phi \approx 0.165$ for the polystyrene particles^57^. For the frequency, $f_h=5\;{\mathrm{MHz}}$ the critical size, $2a_c$, approximately is calculated as $1.24 \;\mu {\mathrm{m}}$.

With a careful attention to the above results, these two distinct regimes can be extracted: (1) Particles much smaller than the critical size, which can be used as a micromixer of suspension fluids. (2) Particles larger than the critical size, which rapidly concentrate at the center of the microchannel. These phenomena can be considered in setups to trap or mix microparticles and biological cells.

### Effects of the sinusoidal boundary amplitudes

**Figure 8:**
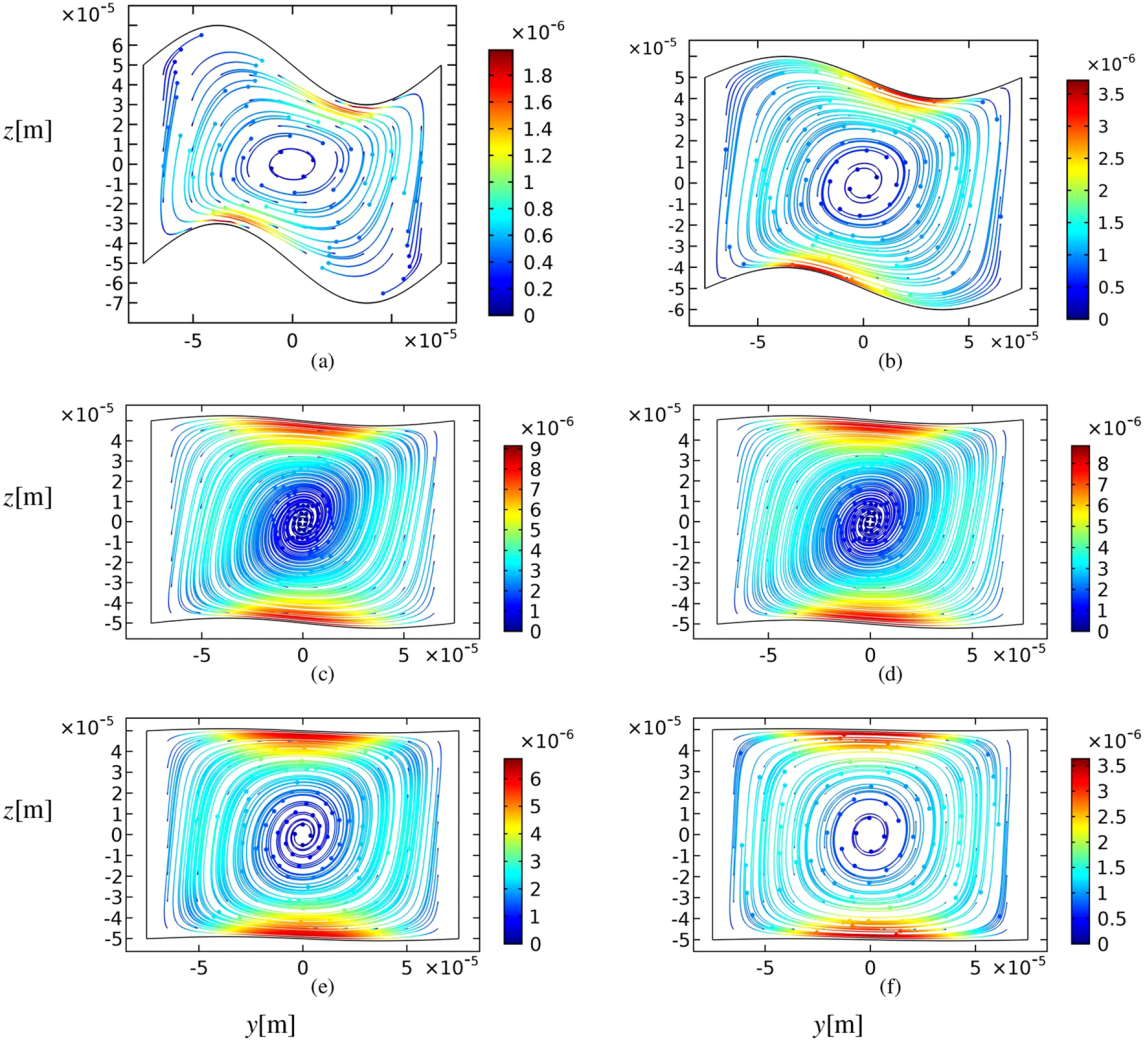
Particle trajectories in m/s for various amplitudes of the sinusoidal boundaries, *A*: (**a**) *h*/5, (**b**) *h*/10, (**c**) *h*/40, (**d**) *h*/50, (**e**) *h*/100 and (**f**) *h*/200. The particle sizes are $d=2\;\mu {\mathrm{m}}$ and the actuation frequency of the top and bottom walls is $f_h$. All the snapshots are illustrated at 100 s. The color contours show $\left\langle \varvec{v}_2 \right\rangle$ values.

**Figure 9:**
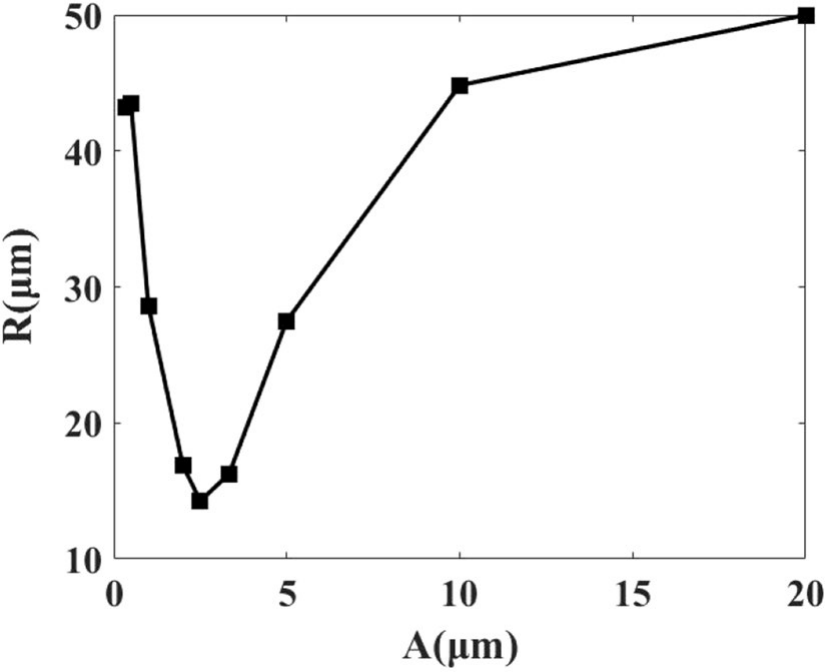
*R* versus *A* in microchannels with $w/h=1.5$, after 100 s. The particles diameter is $d=2\;\mu {\mathrm{m}}$ and the actuation frequency of the top and bottom walls is $f_h$.

In this section, effects of the sinusoidal boundary amplitude, *A*, on particle trajectories are studied. Figure 8 shows snap-shots with various *A* values for particles with $d=2\;\mu {\mathrm{m}}$ at 100 s. The results show that for small and large values of *A* trajectories of particles will be almost aligned, while for intermediate amplitude values, particles will be trapped at the center of the microchannel. In addition, the streaming velocities for intermediate amplitudes are faster. It is noteworthy that there is an optimal value for *A* about 2.5 $\mu m$, in which the particles trapping at the center of the microchannel occurs more efficiently. Figure 9 shows a typical particle distance from the center of the microchannel, *R*, after 100 s for various *A*.

### Effects of the microchannel widths and heights

**Figure 10:**
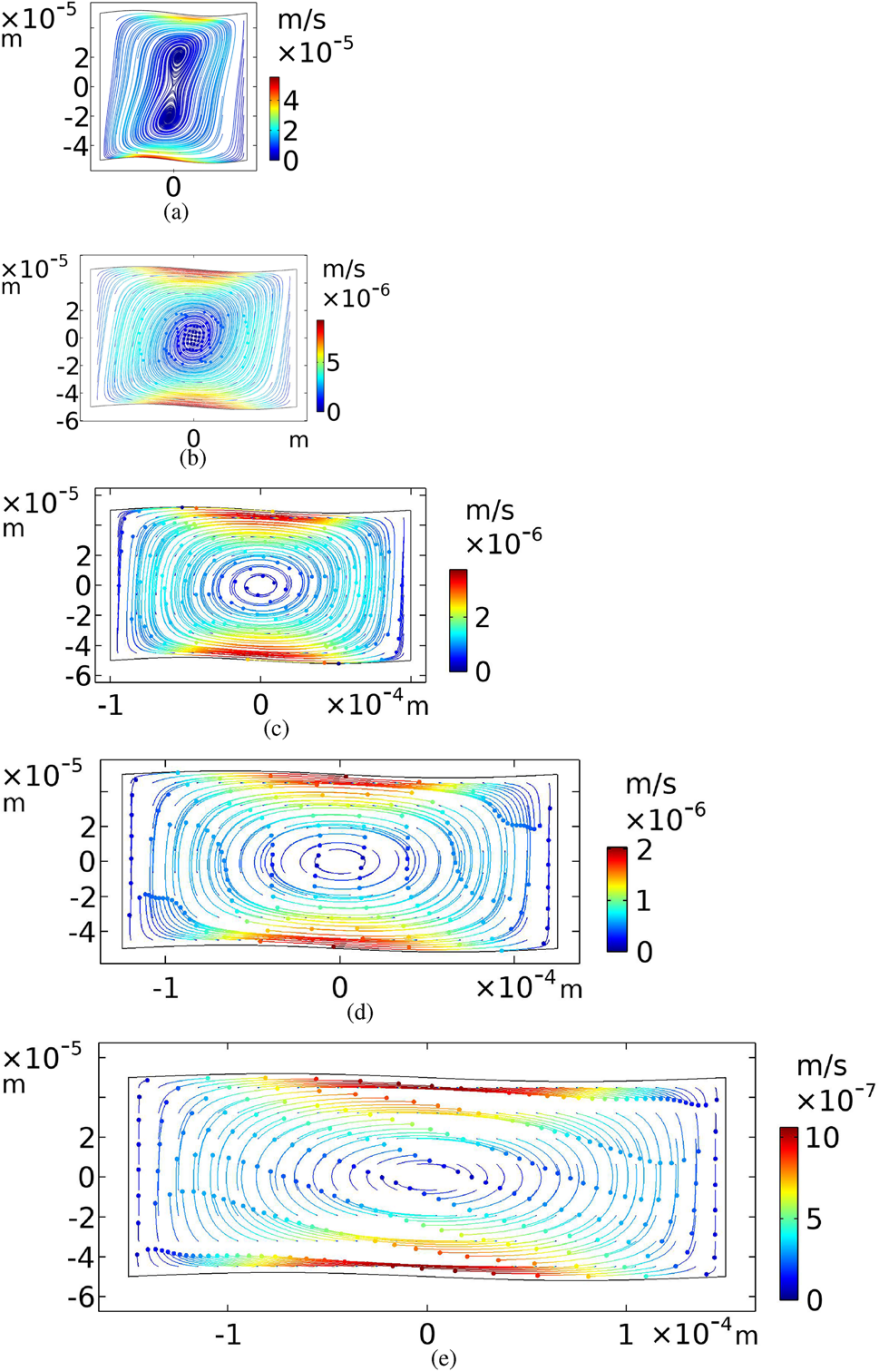
Particle trajectories in m/s for channel widths, *w*, equal to (**a**) 100, (**b**) 150, (**c**) 200, (**d**) 250 and (**e**) $300 \mu {\mathrm{m}}$, and fixed height, $h=100 \mu {\mathrm{m}}$ .The particle sizes are $d=2\;\mu {\mathrm{m}}$, the actuation frequency of the top and bottom walls is $f_h$ and the amplitude of sinusoidal walls is $A = h/50$. All the snapshots are illustrated at 100 s. The color contours show $\left\langle \varvec{v}_2 \right\rangle$ values.

Another investigation has carried out focusing on the different values of microchannel widths. Figure 10 illustrates some examples. Results show that at the fixed vertical oscillation frequency of $f_h=5\;{\mathrm{MHz}}$, spiral paths become less compressed and the concentration of microparticles slows down gradually as a microchannel becomes wider. However, decreasing the width divide the mono vortex into two strong vortices.

**Figure 11:**
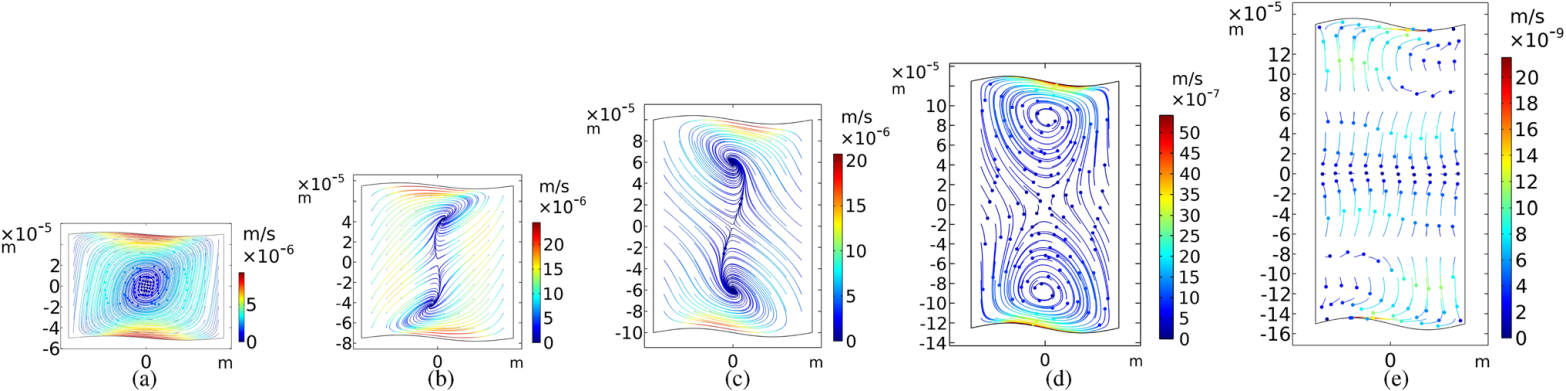
Particle trajectories in m/s for channel heights, *h*, equal to (**a**) 100, (**b**) 150, (**c**) 200, (**d**) 250 and (**e**) 300 $\mu {\mathrm{m}}$, and fixed width, $w=150 \mu {\mathrm{m}}$. The particle sizes are $d=2\;\mu {\mathrm{m}}$, the actuation frequency of the top and bottom walls is $f_h=5$ MHz and the amplitude of sinusoidal walls is $A = h/50$. All the snapshots are illustrated at 100 s. The color contours show $\left\langle \varvec{v}_2 \right\rangle$ values.

The microchannel height is another parameter that worth to be investigated. Our results show that in the fixed vertical oscillation frequency of $f_h=5\;{\mathrm{MHz}}$, as the height of the microchannel is increased, the mono vortex turns into two strong vortices (see Fig. 11a-c). If we continue to increase the height of the microchannel, the trapping effect will be decreased as shown in Fig. 11d,e. Additionally, the movement of particles slows down.

### Square-sinusoidal geometry

**Figure 12:**
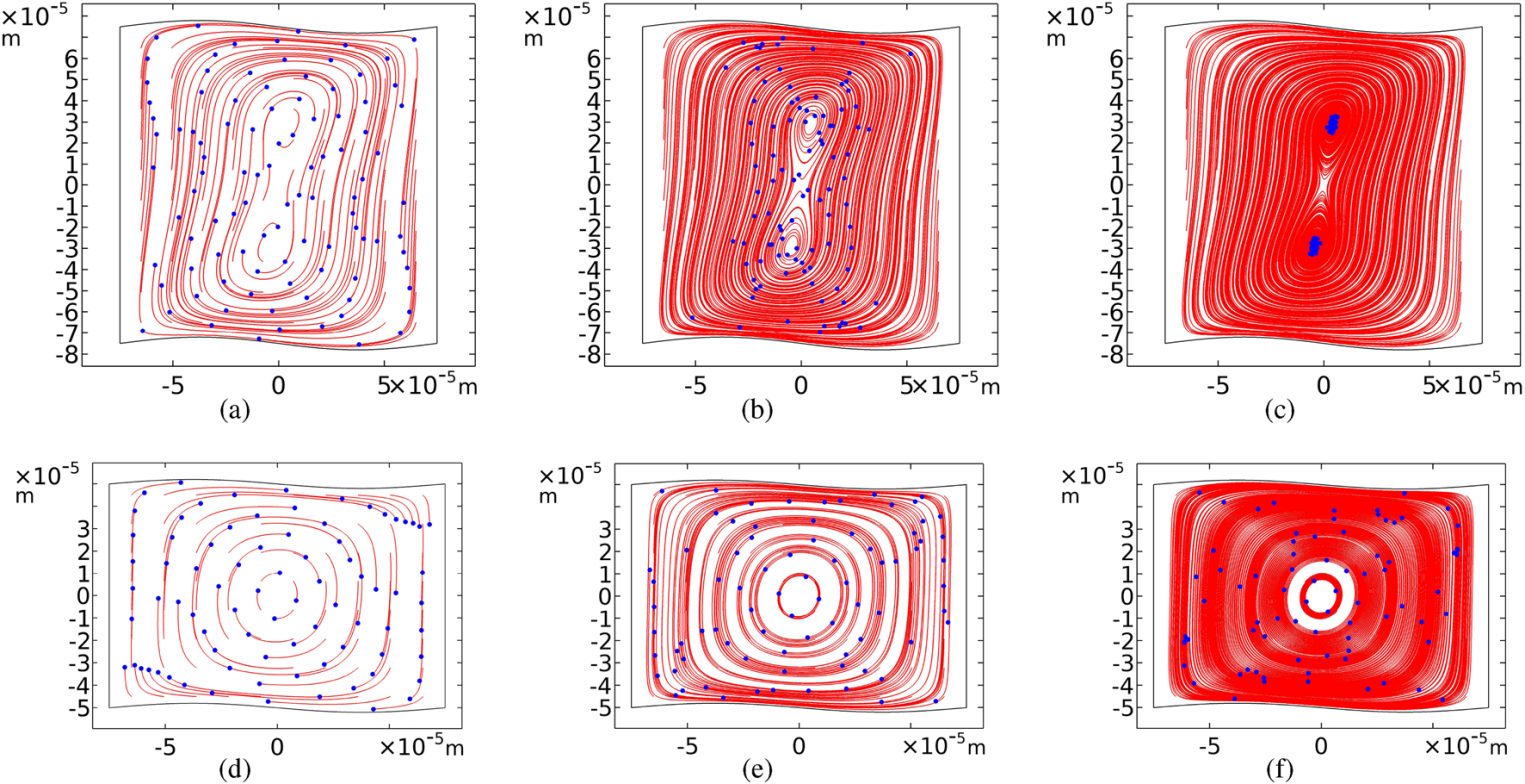
Comparison of the acoustophoretic trajectories of microparticles for the square sinusoidal cross-section (top) and the rectangular ones (bottom). The particles size is $d=0.5\;\mu$m, the amplitude of sinusoidal walls is $A = h/50$ and the actuation frequency of the top and bottom walls is $f_h$. Snapshots are given at (**a**), (**d**) 10, (**b**), (**e**) 60, and (**c**), (**f**) 300 s.

In previous results, we find that in a square-sinusoidal cross-section, two strong vortices emerge instead of one vortex. Figure 12 represents the trajectories for the particles with a radius of $a=250$ nm inside rectangular-sinusoidal and square-sinusoidal cross-sections after definite time scales. The results show that the tiny particles tend to concentrate dramatically faster (about 10 times) in square-sinusoidal geometry than the rectangular ones. Therefore, it is suggested to use square-sinusoidal microchannels for effective trapping of sub-micrometer particles. The particles in a square-sinusoidal cross-section at the frequency of $f_h$ completely aggregate after 300 s.

## Conclusion

In this paper, two-dimensional trajectories of micro-particles inside an acoustically actuated microchannel with sinusoidal top and bottom boundaries have been studied. Our results have shown while the top and bottom walls are vertically oscillating with the horizontal half-wave resonance frequency, an approximately standing acoustic wave is created in the horizontal direction. The resulting acoustic streaming pattern is a large mono-vortex which could never be achieved in a rectangular geometry with flat walls and one-dimensional oscillations. The drag force caused by such vortex inside the microchannel in combination with the tilted acoustic radiation force, leads to trapping of micro-particles with diameters larger than the critical size, after moving on spiral paths. Particles smaller than the critical size, follow close trajectories for a long time. For larger particles, the acoustic radiation force is dominant, which makes particles to be trapped. Whereas, for smaller particles it became negligible, but the acoustic streaming drag force is dominant.

In addition, the simulation results have shown that for small and large amplitudes of sinusoidal walls trajectories of particles would eventually be aligned, while for intermediate amplitudes the particles would be trapped at the center of the microchannel.

Moreover, results of the square sinusoidal geometry analysis have shown that there are two strong vortices in this case instead of one vortex. In this setup the tiny particles tend to concentrate dramatically faster than the rectangular sinusoidal geometry.
